# Attitudes of Non-psychiatric Doctors Toward the Management of Psychiatric Problems

**DOI:** 10.7759/cureus.47229

**Published:** 2023-10-17

**Authors:** Gautami Nagabhirava, Saradhi Goud, Akhil D Goel

**Affiliations:** 1 Psychiatry, Kamineni Academy of Medical Sciences and Research Center, Hyderabad, IND; 2 Community and Family Medicine, All India Institute of Medical Sciences, Jodhpur, Jodhpur, IND

**Keywords:** collaborative care, psychiatric symptoms, health care professionals’ attitudes, mental healthcare, consultation liaison psychiatry, general hospital doctors, psychosocial problems

## Abstract

Introduction: This research explores non-psychiatric doctors' attitudes toward managing psychiatric problems, recognizing the critical intersection between physical and emotional health. The study aims to understand the barriers and facilitators in addressing these challenges within a tertiary care hospital in Hyderabad, India. The prevalence of psychiatric disorders among general hospital inpatients and outpatients underscores the need for comprehensive care. However, various obstacles hinder effective management. The objectives are to describe and understand these attitudes and to investigate the reasons for non-referral in cases involving psychiatric concerns.

Methods: A cross-sectional study was conducted from April to May 2023, involving 178 doctors from various specialties directly engaged in patient care. Participants completed a modified Doctors Attitudes Toward Collaborative Care for Mental Health (DACC-MH) questionnaire. This tool assessed their attitudes toward psychosocial and psychiatric problems, including their willingness to take responsibility for assessments and referrals. Data analysis utilized the IBM Statistical Package for the Social Sciences (SPSS) Version 22 (IBM Corp., Armonk, NY). Descriptive statistics and chi-square tests were used to assess differences in attitudes based on demographics and specialties.

Results: The study revealed predominantly positive attitudes among non-psychiatric doctors. Most acknowledged the importance of addressing patients' emotional problems (97.8%) and recognizing psychological factors' role in physical illnesses (96.1%). However, variations existed in the willingness to take responsibility for psychological assessments, especially in outpatient settings. Attitudes toward psychiatric referrals were generally positive, though differences were noted based on gender and specialization. Female doctors were more inclined toward emotional care, while male doctors were more willing to prescribe psychotropic drugs (p < 0.0001) and refer patients to psychiatrists. Physicians were more favorable toward emotional care, shared responsibility for emotional difficulties, and routine assessment of psychological and social factors. In contrast, surgical specialists restricted themselves to physical assessments (p < 0.0001).

Conclusion: This research underscores the need for targeted educational initiatives and awareness campaigns to address the challenges in integrating mental healthcare into general healthcare contexts. Tailored programs, interprofessional collaborations, and efforts to reduce stigma are essential for improving doctors' attitudes and practices in managing psychiatric problems. Enhancing the integration of mental health care can lead to better patient outcomes and overall healthcare quality. Healthcare institutions can strive for more comprehensive, patient-centered care by understanding and addressing these attitudes.

## Introduction

There is a strong connection between physical and emotional health, and it is common to find psychiatric disorders among general hospital inpatients and outpatients [1]. Psychological factors, such as chronic stress, poor psychological functioning, and mental health disorders, often contribute to the development of physical conditions, including obesity, diabetes, chronic heart disease, and cardiovascular disease [2]. Unfortunately, many patients lack knowledge and awareness of mental health issues, often mistaking their somatic symptoms, anxiety, and depression as physical ailments. This poses a challenge as non-psychiatric doctors are typically considered; however, they may overlook or delay the diagnosis, treatment, or referral of patients with mental health concerns despite the high prevalence and comorbidity [3]. Non-psychiatric doctors often contend with the dual challenge of limited time, which can hinder their ability to consider patients' psychological aspects, and the need for further training, all while managing their demanding schedules, leaving little room for upskilling. Moreover, the societal stigma surrounding mental illness discourages patients and doctors from seeking and providing appropriate help [4]. This situation leads to further complications, including treatment delays, poorer patient outcomes, increased hospital admission rates, readmission, and ultimately higher healthcare utilization [3].

Addressing these challenges mandates the proactive involvement of non-psychiatric doctors in the comprehensive management of psychiatric issues. Timely referrals to specialized psychiatric care, establishing consultation-liaison psychiatry, and optimizing clinical outcomes are imperative [5]. However, even as the concept of consultation-liaison psychiatry gains traction within India, attitudinal barriers toward psychiatry's integration into broader healthcare contexts can hinder effective collaboration and the provision of comprehensive care [6]. This study seeks to describe and understand attitudes held by non-psychiatric doctors toward psychosocial and psychiatric problems in a tertiary care hospital in Hyderabad.

Objectives

The objectives are to describe and understand the general attitudes held by non-psychiatric doctors toward psychosocial and psychiatric problems and their management and to explore the underlying reasons contributing to non-referral in cases involving psychiatric concerns.

## Materials and methods

This cross-sectional study aims to assess the attitude of non-psychiatric doctors toward psychiatric and psychosocial problems. Institutional ethical committee approval was obtained on March 16, 2023 from the Kamineni Academy of Medical Sciences and Research Center before the initiation of the study (#EC/NEW/INST/2021/1676). Data collection was conducted from April to May 2023 through a convenient sampling method. During this period, a qualified psychiatrist made visits to all clinical departments. Departments with little direct patient care were excluded from the study. At the commencement of these visits, participants were provided with a concise introduction to the study's objectives and methodology. Written informed consent was obtained from each participant before they were given the questionnaire. After the completion of visits, a follow-up phase was initiated. An online questionnaire was prepared and hosted on the Google Forms platform. An email containing a reminder and a link to the online questionnaire was distributed to all eligible participants.

Inclusion criteria

Consultants of eligible departments who are directly involved with patient care and residents of eligible departments directly engaged in patient care responsibilities were included. 

Exclusion criteria 

Excluded were consultants/departments with minimal or no patient care responsibilities and interns. 

Instruments 

For this study, a modified version of the Doctors Attitudes toward Collaborative Care for Mental Health (DACC-MH) Scale, a questionnaire for evaluating attitudes of non-psychiatric doctors towards mental health problems and Consultation Liaison services, developed by I. Wilamaratne, was employed [7,8]. Non-psychiatric doctors' attitudes toward assessing, referring, and managing psychological problems were assessed through this 42-item questionnaire. Furthermore, other demographic details, such as age, gender, specialty, and years since qualifying as a specialist, were collected. The questionnaire is typically completed in approximately 10 minutes.

Statistical analysis

The data analysis process involved the utilization of IBM Statistical Package for the Social Sciences (SPSS) Statistics, Version 22 (IBM Corp., Armonk, NY, USA). All statistical tests were conducted with a two-tailed approach, and a significance level of 0.05 (alpha = 0.05) was employed.

Descriptive statistics provided a comprehensive overview of participants' responses, allowing us to record the percentage of agreement with each questionnaire item. Furthermore, chi-square tests were utilized to discern variations and disparities within subgroups, such as those based on demographic characteristics and medical specialties. This statistical method was chosen for its effectiveness in analyzing categorical data and identifying significant differences among distinct segments of the study participants.

## Results

Sample description

A total of 178 doctors willingly participated in this questionnaire. The demographic analysis revealed a nearly equal distribution between genders, with 51.1% male and 48.9% female. Respondents were further categorized into distinct age groups and years since qualifying or trainee, with the majority falling within the age group of under 30 years (45.5%), while half of the doctors were identified as resident trainees (51.1%). A detailed presentation of the demographic characteristics, encompassing gender, age, and years since qualifying as specialists, is provided in Table 1.

**Table 1:** Demographic characteristics of respondents

Categories		Number (n)	Percentage (%)
Gender	Male	91	51.1
	Female	87	48.9
Age	<30 years	81	45.5
	30-39 years	45	25.3
	40-49 years	27	15.2
	50-59 years	15	8.4
	>60 years	10	5.6
Speciality	Physicians	90	50.6
	Surgeons	88	49.4
Years since qualifying as a specialist/Title	Resident Trainee	91	51.1
	< 5 years	17	9.6
	5-9 years	16	9.0
	10-19 years	32	18.0
	>20 years	22	12.4

General attitudes

Attitudes were predominantly positive, with most respondents acknowledging that dealing with patients' emotional problems is integral to a doctor's work (97.8%). Respondents also agreed that psychological factors are important in the course of physical illness (96.1%). However, a variation in willingness to take responsibility manifested in differing levels of enthusiasm for psychological assessments for inpatients (89.3%) and outpatients (44.4%). Interestingly, 38.2% of the respondents believed that emotional and social factors did not enhance their job interests.

Views also differed on sharing responsibilities for managing social and emotional difficulties among inpatients, with the majority advocating for shared responsibility (93.3%). Respondents also leaned towards viewing it as a primary medical (57.9%) or nursing (26.4%) duty. Furthermore, more than half of the respondents (58.4%) believed that the emotional care of patients by hospital doctors is impractical under present conditions. Surprisingly, 65.2% of the respondents disagreed that general practitioners are primarily responsible for assessing emotional problems in new outpatients.

Attitudes to treatment 

Despite 51.1% of the respondents believing that hospital doctors should be able to prescribe psychotropic drugs, only 21.3% reported regular prescriptions of antidepressants. 54.5% believe hospital doctors should be able to discuss psychological problems with relatives; however, only 29.2% of respondents frequently discuss patients' emotional problems with relatives. It is noteworthy that a substantial portion of respondents (32.0%) admit to confining themselves to physical assessments when psychological factors are a significant contributing factor (Table 2).

**Table 2:** Questionnaire ( Modified DACC-MH)* and percentage in agreement *Doctors Attitudes toward Collaborative Care for Mental Health

Question	Agree (%)	Disagree (%)
Dealing with patients’ emotional problems is part of the hospital doctors’ work	97.8	2.2
Management of social and emotional difficulties of inpatients is primarily a medical responsibility.	57.9	42.1
Management of social and emotional difficulties of inpatients is primarily a nursing responsibility.	26.4	73.6
Management of social and emotional difficulties of inpatients is a shared responsibility.	93.3	6.7
The variety of patients’ emotional and social care enhances my job interest.	61.8	38.2
Psychological factors are important in the course of physical illness.	96.1	3.9
Management of emotional problems is an important part of my care of chronic outpatients.	71.9	28.1
Emotional care of patients by hospital doctors is impractical under present conditions.	58.4	41.6
General practitioners are primarily responsible for assessment of emotional problems in new outpatients.	34.8	65.2
Psychological and social factors should be routinely assessed and recorded for inpatients	89.3	10.7
Psychological and social factors should be routinely assessed and recorded for outpatients.	44.4	55.6
Hospital doctors are not responsible for emotional care of patients.	16.3	83.7
I should concern myself with emotional care of patients with chronic physical illnesses.	84.3	15.7
I Prescribe antidepressants frequently/occasionally.	21.3	78.7
Hospital doctors should be able to prescribe psychotropic drug treatments	51.1	48.9
I frequently discuss patients’ emotional problems with relatives	29.2	70.8
When psychological factors appear to be an important cause of the presenting problem, I confine myself to physical assessment	32.0	68.0
Hospital doctors’ should be able to use psychological methods such as listening/reassurance	100	0
Hospital doctors’ should be able to use psychological methods like discussion of anxiety/problems	92.1	7.9
Hospital doctors should be able to discuss patients’ psychological problems with relatives	54.5	45.5
Hospital doctors’ should be able to use cognitive or behavioral treatments	79.8	20.2
I would welcome more contact with psychiatrists	98.9	1.1
I would consider referring a patient with depression to psychiatrists	99.4	0.6
I would consider referring a patient with disturbed behavior to psychiatrists	96.1	3.9
I would consider referring a patient with diagnostic problems to psychiatrists	48.9	50.6
I would consider referring a patient with treatment non-compliance to psychiatrists	59.0	41.0
I would consider referring a patient with disturbed dementia to psychiatrists	82.0	18.0
I would consider referring a patient with acute confusional state to psychiatrists	88.2	11.8
Patient is disadvantaged by being labelled as psychiatric case	43.8	56.2
Psychiatric service is dissatisfactory	10.1	89.9
Psychiatric disorders are incurable.	7.3	92.7
Psychiatric treatments are ineffective	5.1	94.9
Patients dislike psychiatric referral	93.8	6.2
Patients Refuse Psychiatric Referral	86.5	13.5
I do not know the patient well enough to refer	25.8	74.2
Patients are too physically ill to be referred to psychiatry.	28.1	71.9
I would like to know more about what psychiatrists have to offer in the management of medical and surgical patients.	94.9	5.1
I would like more contact with the psychiatric service	99.4	0.6
Psychiatrists have little to offer in a general hospital	7.3	92.7
I would welcome more non- medical help in the management of patients’ emotional problems.	75.8	24.2
I would welcome more time to talk to my patients	93.3	6.7
I would like more help in providing psychological and social care	94.4	5.6

Attitudes to referral

When inquired about their views on psychiatric referrals, over 95% of the participants indicated their willingness to recommend psychiatric referrals for patients with depression (99.4%) and disturbed behavior (96.1%). Many doctors were open to referring patients experiencing acute confusion (88.2%) and disruptive dementia (82%). However, a smaller percentage, around 59%, would consider a psychiatric referral for noncompliance, and fewer than half, precisely 48.9%, would do so for patients facing complex diagnostic challenges. While a minority of respondents held beliefs that psychiatric services or treatments were unsatisfactory (10.1%), incurable (7.3%), and ineffective (5.1%), it is noteworthy that a majority of participants shared the consensus that patients generally harbor an aversion towards psychiatric referrals (93.8%) and often exhibit reluctance toward undergoing the prescribed treatment (86.5%). One-fourth of the respondents reported not knowing the patient well enough to refer to psychiatry (25.8%).

Attitudes to needs in practice

Nearly all of the respondents expressed a strong desire for increased interaction with psychiatrists, with an overwhelming 99.4% indicating their openness to greater contact. Moreover, a substantial majority, accounting for 94.9% of the participants, expressed keen interest in understanding the contributions and services that psychiatrists could offer. In addition to their desire for enhanced collaboration with psychiatric professionals, the respondents also conveyed a significant appetite for more time dedicated to patient communication, with 93.3% endorsing the need for extended patient-doctor interactions. Furthermore, a noteworthy 94.4% of the participants expressed their inclination to receive additional support in delivering psychological and social care to their patients.

Difference between physicians and surgeons

We grouped the respondents further into physicians (Pediatrics, General Medicine, Neurology, Cardiology, Endocrinology, Dermatology, Pulmonology, Gastroenterology, Nephrology) and surgeons (General Surgery, Ophthalmology, Cardiothoracic Surgery, Obs/Gyn, Orthopedics, Urology, ENT, and Plastic Surgery). 50.6% (n=90) and 49.4% (n=88) of the respondents were grouped into physician and surgeon groups, respectively (Table 1). Chi-square test was used for statistical analysis.

Overall, physicians had a better attitude than surgeons regarding the importance of psychological health, such as psychological factors are important during physical illness (p = 0.006). More physicians reported that the variety of patients' emotional and social care enhanced their job interests (p = 0.0231). 

Medical specialists were more likely to believe that dealing with patient's emotional problems is part of the hospital doctor's work compared to surgeons (p = 0.0414). The willingness to take responsibility differed, with surgeons believing that managing the social and emotional difficulties of inpatients is primarily a nursing responsibility compared to physicians (p = 0.0009). Meanwhile, more physicians reported believing that it is a shared responsibility (p = 0.0153). Physicians were more likely to believe that psychological and social factors should be routinely assessed and recorded for inpatients (p = 0.0066).

More physicians believed that hospital doctors should be able to discuss patients' problems with relatives (p = 0.0169), should be able to prescribe psychotropic drug treatments (p < 0.0001), should be able to use cognitive or behavioral treatments (p = 0.0001), use psychological methods like discussion of anxiety problems (p = 0.0007). They were also more likely to prescribe antidepressants (p = 0.0045).

On the other hand, when psychological factors appeared to be an important cause of presenting problems, more surgeons confined themselves to physical assessment compared to physicians (p < 0.0001). Surgeons were also less likely to refer patients with disturbed dementia (p = 0.0437). They also report not knowing the patients enough to refer them (p = 0.0326) compared to their counterparts. Conversely, more physicians reported that patients are sometimes too physically ill to be referred to psychiatry (p = 0.0012). Physicians reported the willingness to receive more medical help in the management of patient's emotional problems (p = 0.0023). Surgeons reported wanting to know more about what psychiatrists have to offer in the management of medical and surgical patients (p = 0.0024).

Difference in gender

When examining gender differences, distinct patterns emerged in various medical care perceptions. Female respondents were more inclined to perceive emotional care as an integral part of a doctor's role compared to males (p = 0.0486). Additionally, more females acknowledged the importance of managing emotional problems in caring for chronic outpatients than males (p = 0.0017).

Conversely, more males believed that hospital doctors should be able to prescribe psychotropic drugs (p = 0.0006). This tendency was reflected in actual practice, with 35.2% of males regularly prescribing antidepressants, contrasting with only 6.9% of females (p < 0.0001). Gender disparities extend to views on patients labeled as psychiatric cases; males were more likely to perceive patient disadvantage due to labeling (p = 0.0023).

Male doctors exhibited a greater willingness to refer patients with diagnostic and dementia-related issues to psychiatrists compared to female doctors (p = 0.0083 and p = 0.0369, respectively.) On the other hand, male doctors also held the opinion that patients could sometimes be too physically ill for psychiatric referral (p = 0.0005) and that patients might decline psychiatric referrals (p = 0.0211). Moreover, male respondents were more inclined to view psychiatric services as unsatisfactory or ineffective (p = 0.0040 and p = 0.0027, respectively).

## Discussion

Often the point of first contact, the decision to include postgraduate residents in our study was grounded in recognition of the substantial burden placed on these students in Indian hospitals, owing to high patient loads. This inclusion differentiates our study from others which excluded these students [9]. Overall, we found positive attitudes toward psychiatric and psychosocial problems among non-psychiatric doctors. Most respondents acknowledged that dealing with patients' emotional problems is part of a doctor's work and that psychological factors are important during physical illness. These results align with previous literature, such as Nauta et al. [10] and Wang et al. [11], and underscore the evolving understanding within the medical community that patient well-being encompasses physical and psychological dimensions. This sentiment has been notably emphasized in a cross-sectional survey conducted across seven countries [9].

Despite this understanding, similar to studies in the Netherlands [10], China [11], and London [7], our study also revealed discrepancies in the practical implementation of psychosocial care, with a significant portion of our respondents expressing doubt regarding the feasibility of emotional care in present conditions. The finding that some doctors did not believe that emotional and social factors enhanced their job interest suggests room for improvement in emphasizing the holistic care of patients.

The study's findings concerning attitudes toward treatment revealed a notable disparity between doctors' beliefs and their actual clinical practices. In our study, there was a prevailing belief that hospital doctors should be able to prescribe psychotropic drugs (51.1%); however, this ideal was not consistently reflected in real-world prescription practices, particularly concerning antidepressants (21.3%). Likewise, the conviction that doctors should discuss psychological issues with patients' relatives (54.5%) only sometimes translated into such discussions occurring regularly in practice (29.2%). Perhaps most strikingly, a significant proportion of doctors (32%) limited their assessments to physical aspects when confronted with patients presenting significant psychological factors. This is higher than the 23.5% in a study by Nauta et al. [10] and 16% by Morgan et al. [7].

Most participants were willing to recommend psychiatric referrals for patients with depression and disturbed behavior. The lower inclination among doctors to refer patients with dementia, treatment non-compliance, and diagnostic problems to psychiatrists may be attributed to the perception that these issues fall under the expertise of neurology and other specialized departments. However, this perspective is problematic for several reasons. Firstly, it overlooks the high prevalence of behavioral and psychological symptoms in dementia patients. These symptoms often require a comprehensive approach combining neurological and psychiatric expertise. Additionally, conditions like treatment non-compliance and complex diagnostic problems can have underlying psychological aspects and potential somatic underpinnings. Neglecting the possible co-occurrence of psychiatric illnesses reflects inadequate awareness and knowledge among doctors.

Furthermore, our study revealed that most respondents reported that patients tend to refuse or dislike psychiatric treatment. The finding suggests that some doctors may refrain from making psychiatric referrals out of concern for stigmatizing patients or fear of losing them as clients. Targeted educational initiatives and awareness campaigns are essential to address these challenges effectively. Such efforts can help healthcare professionals better understand the nuanced nature of these conditions and the importance of collaborative care in improving patient outcomes. Our study found that respondents were more inclined to conduct psychological assessments for inpatients than outpatients. This could be attributed to time constraints and lack of skills, issues noted in previous studies such as Wang et al. [11]. This finding underscores the importance of providing doctors with the necessary training and resources to address psychosocial issues effectively in outpatient settings.

Specialization differences highlighted that physicians exhibited more positive attitudes towards psychological health and care than surgeons. Physicians were more likely to view emotional care as part of a doctor's role, believed in shared responsibility for emotional difficulties, and advocated for routine assessment of psychological and social factors. This difference was found in some studies in China [11], Canada [12], Netherlands [10] but not in Tehran [13] and London [7], where no significant correlation was found.

Gender differences were also observed, with females more likely to view emotional care as integral to a doctor's role. This is in concordance with previous literature such as Wang et al. [11], Nauta et al. [10], and Morgan et al. [7]. On the other hand, males were more likely to believe that doctors should be able to prescribe psychotropic drugs and confessed to doing so regularly. Males were also more likely to refer patients with diagnostic and dementia-related concerns to psychiatrists, albeit accompanied by reservations about their physical well-being, potential refusal of psychiatric referrals, and the disadvantage of labeling. This difference in approach regarding management is possibly due to males being more action-oriented or using more heuristics than females, which has been noted in previous studies such as Nauta et al. [10], Lagro et al. [14], and Gotlieb et al. [15]. Additionally, male respondents were more inclined to hold critical views on the effectiveness of psychiatric services.

Strengths and implications

This study's strengths include its diverse representation, encompassing postgraduate residents for insights into early-career doctors' experiences. It offers comprehensive demographic data, a substantial sample size, and distinguishes between physicians and surgeons. Gender-based disparities in healthcare perceptions are also explored. Despite predominantly positive attitudes toward holistic healthcare, a notable gap exists in practical implementation, particularly in psychotropic drug prescriptions and psychiatric referrals. These findings highlight the need for tailored training programs to address gender and specialization-based differences among doctors. Additionally, the study underscores the importance of combating stigmatization concerns and ensuring adequate resources for managing psychosocial issues to enhance patient care in clinical settings.

Limitations

The study's use of a convenient sampling method indicates a potential sampling bias attributed to the specific time frame of data collection, meaning certain doctors could be excluded due to their varying schedules or availability. Participant responses could be influenced by social desirability bias, and the presence of a qualified psychiatrist might inadvertently introduce bias as participants might feel pressured to align their responses with perceived expectations. The study's focus on a single tertiary care hospital may limit the generalization of the findings. Excluding departments with minimal patient care involvement may introduce selection bias, omitting valuable perspectives from non-patient care departments.

## Conclusions

This study reveals that non-psychiatric doctors generally hold positive attitudes towards psychosocial and psychiatric issues, but challenges hinder the integration of mental health care into general healthcare settings. Variations in their willingness to address psychological issues in different patient settings underscore the need for targeted training programs. There is also a gap between doctors' belief in their capacity to prescribe psychotropic drugs and their actual prescription rates, indicating barriers to effective medication use. The enduring stigma around psychiatric referrals and treatments emphasizes the importance of destigmatization efforts. Proactive measures such as tailored education and interprofessional collaborations are crucial to improving doctors' attitudes and practices in managing psychiatric problems for better patient outcomes and a more holistic healthcare approach. Future research should focus on longitudinal, qualitative, and intervention studies and explore the impact of stigma reduction strategies to enhance mental health integration in healthcare.

## References

[1] van Niekerk M, Walker J, Hobbs H (2022). The prevalence of psychiatric disorders in general hospital inpatients: a systematic umbrella review. J Acad Consult Liaison Psychiatry.

[2] Levine GN, Cohen BE, Commodore-Mensah Y (2021). Psychological health, well-being, and the mind-heart-body connection: a scientific statement from the American Heart Association. Circulation.

[3] Kroenke K, Unutzer J (2017). Closing the false divide: sustainable approaches to integrating mental health services into primary care. J Gen Intern Med.

[4] Henderson C, Evans-Lacko S, Thornicroft G (2013). Mental illness stigma, help seeking, and public health programs. Am J Public Health.

[5] Wood R, Wand APF (2014). The effectiveness of consultation-liaison psychiatry in the general hospital setting: Aa systematic review. J Psychosom Res.

[6] Grover S, Avasthi A (2019). Consultation-liaison psychiatry in India: where to go from here?. Indian J Psychiatry.

[7] Morgan JF, Killoughery M (2003). Hospital doctors' management of psychological problems - Mayou & Smith revisited. Br J Psychiatry.

[8] Mayou R, Smith EB (1986). Hospital doctors' management of psychological problems. Br J Psychiatry.

[9] Wimalaratne IK, Mccarthy J (2021). General hospital specialists’ attitudes toward psychiatry: a cross-sectional survey in seven countries. BMJ.

[10] Nauta K, Boenink AD, Wimalaratne IK, Menkes DB, Mellsop G, Broekman B (2019). Attitudes of general hospital consultants towards psychosocial and psychiatric problems in Netherlands. Psychol Health Med.

[11] Wang J, Wang Q, Wimalaratne I, Menkes DB, Wang X (2017). Chinese non-psychiatric hospital doctors' attitudes toward management of psychological/psychiatric problems. BMC Health Serv Res.

[12] Thombs BD, Adeponle AB, Kirmayer LJ, Morgan JF (2010). A brief scale to assess hospital doctors' attitudes toward collaborative care for mental health. Can J Psychiatry.

[13] Hamdieh M, Banihashem S, Beyraghi N, Abbasinejad M, Hagh-Ranjbar F (2015). Physicians' attitudes toward integrating consultation-liaison psychiatric services in four major general hospitals in Tehran. Gen Hosp Psychiatry.

[14] Lagro-Janssen AL (2008). Medicine is not gender-neutral: influence of physician sex on medical care (Article in Dutch). Ned Tijdschr Geneeskd.

[15] Gotlieb R, Abitbol J, How JA (2019). Gender differences in how physicians access and process information. Gynecol Oncol Rep.

